# The RNA Helicase BELLE Is Involved in Circadian Rhythmicity and in Transposons Regulation in *Drosophila melanogaster*

**DOI:** 10.3389/fphys.2019.00133

**Published:** 2019-02-20

**Authors:** Paola Cusumano, Milena Damulewicz, Elena Carbognin, Laura Caccin, Antonietta Puricella, Valeria Specchia, Maria Pia Bozzetti, Rodolfo Costa, Gabriella M. Mazzotta

**Affiliations:** ^1^Department of Biology, University of Padua, Padua, Italy; ^2^Department of Cell Biology and Imaging, Jagiellonian University, Kraków, Poland; ^3^Department of Biological and Environmental Sciences and Technologies, University of Salento, Lecce, Italy

**Keywords:** RNA helicase BELLE, *Drosophila*, post-transcriptional control, circadian clock, piRNA-mediated transposons regulation

## Abstract

Circadian clocks control and synchronize biological rhythms of several behavioral and physiological phenomena in most, if not all, organisms. Rhythm generation relies on molecular auto-regulatory oscillations of interlocked transcriptional-translational feedback loops. Rhythmic clock-gene expression is at the base of rhythmic protein accumulation, though post-transcriptional and post-translational mechanisms have evolved to adjust and consolidate the proper pace of the clock. In *Drosophila*, BELLE, a conserved DEAD-box RNA helicase playing important roles in reproductive capacity, is involved in the small RNA-mediated regulation associated to the piRNA pathway. Here, we report that BELLE is implicated in the circadian rhythmicity and in the regulation of endogenous transposable elements (TEs) in both nervous system and gonads. We suggest that BELLE acts as important element in the piRNA-mediated regulation of the TEs and raise the hypothesis that this specific regulation could represent another level of post-transcriptional control adopted by the clock to ensure the proper rhythmicity.

## Introduction

Circadian clocks have evolved in most, if not all, organisms to time their physiology and behavior to the most appropriate phases of the day–night cycles (Young and Kay, 2001; Dubruille and Emery, 2008; Peschel and Helfrich-Forster, 2011). They control and synchronize the biological rhythms of a vast array of behavioral and physiological phenomena, including visual sensitivity, hormone secretion, locomotion, hatching, eclosion, ecdysteroid synthesis, sperm release, gene expression, and sleep wake cycle (Sandrelli et al., 2008; Allada and Chung, 2010; Schiesari et al., 2011). Biological clocks are self-sustained in the absence of environmental cues. Rhythm generation occurs in single cells and is based on molecular auto-regulatory oscillations of interlocked transcriptional-translational feedback loops. In *Drosophila*, the transcription factors CLOCK (CLK) and CYCLE (CYC) activate, as heterodimer, the expression of the clock genes *period* (*per*) and *timeless* (*tim*). The correspondent proteins, PER and TIM, are post-translational regulated and eventually accumulate in the cytoplasm and enter the nucleus, where they ultimately inhibit their own transcription by repressing CLK-CYC activity. Two other interlocked feedback loops regulating the expression of the *Clock* gene and CLK/CYC mediated transcription, respectively, contribute to the robustness of the circadian oscillations (reviewed in Ozkaya and Rosato, 2012).

Rhythmic clock-gene expression is important to generate rhythmic protein accumulation; however, cycling protein levels do not depend only on cycling mRNAs but post-transcriptional and post-translational mechanisms have also evolved to adjust and consolidate the proper pace of the clock (Kojima et al., 2011). Among these, alternative splicing, mRNA nuclear export, polyadenilation, regulation of translation and degradation, play fundamental roles in the post-transcriptional regulation of circadian gene expression (Majercak et al., 1999, 2004; Collins et al., 2004; Boothroyd et al., 2007; Kojima et al., 2012; Huang et al., 2013; MacGregor et al., 2013; Robles et al., 2014; Montelli et al., 2015).

In recent years, a role for small, non-coding RNAs (miRNA) as post-transcriptional regulators of circadian rhythmicity in *Drosophila* has been also established. They regulate the proper development and maintenance of circadian rhythms acting either directly on the expression of clock genes or indirectly on signaling outputs (reviewed in Xue and Zhang, 2018).

Another class of small RNA, the piRNAs, has recently been associated to the age-dependent rhythmicity (Kuintzle et al., 2017). In particular, putative primary piRNA transcripts corresponding to transposons exhibit a *de novo* oscillation in old flies and circadian clock components appear to regulate these late rhythmicity (Kuintzle et al., 2017). piRNAs are small molecules protecting animal cells from the de-regulation of transposons and other repetitive genetic elements, preserving genome stability (Li et al., 2009; Thomson and Lin, 2009; Le Thomas et al., 2014). The piRNA biogenesis starts with the transcription of genomic clusters dispersed in the genome, producing the primary piRNA precursor transcripts (Brennecke et al., 2007). The piRNA pathway was first discovered in the gonads (Vagin et al., 2006; Aravin et al., 2007; Brennecke et al., 2007; Specchia et al., 2008, 2010, 2017; Specchia and Bozzetti, 2009; Bozzetti et al., 2015; Iwasaki et al., 2015; Sahin et al., 2016), but later it was observed in the nervous system of both *Drosophila* and mice (Lee et al., 2011; Perrat et al., 2013).

*belle* emerged in a screen for genes involved in the RNAi pathway: it binds to Ago1 and Ago2, involved in the small RNA-mediated regulation (Kim et al., 2005; Ulvila et al., 2006; Zhou et al., 2008). Very recently, a role of *belle* in the regulation on specific P-derived constructs in the ovary has been described (Lo et al., 2016), and therefore it is now considered a “bona fide” piRNA gene. *belle* encodes a DEAD-box RNA helicase: it is the closest paralog of *vasa* and plays roles in the reproductive system allowing fertility in *Drosophila melanogaster.* Like other members of the DEAD box family, Vasa and BELLE are supposed to have ATPase, RNA binding, and RNA unwinding activities (Liang et al., 1994; Sengoku et al., 2006). BELLE is essential for both male and female reproductive capacity and its function in gonads is conserved during evolution. It localizes at the perinuclear region, called “nuage,” of the germ cells in male and female gonads (Johnstone et al., 2005; Kibanov et al., 2011), where the majority of key components regulating transposable elements (TEs) are located (Lim and Kai, 2007; Klattenhoff and Theurkauf, 2008; Kibanov et al., 2011; Nagao et al., 2011).

Here, we have identified for BELLE important roles as putative circadian clock component and regulator of TEs in brain and in gonads. These apparently unrelated functions suggest the hypothesis that BELLE might be a key element in small RNA (piRNA)-mediated regulation of the TEs and that this regulatory pathway is involved in circadian rhythmicity.

## Materials and Methods

### Fly Strains

The following strains of *Drosophila melanogaster* were used: *w^1118^, CantonS, yw;tim-*Gal4 (Emery et al., 1998), *UAS-belle*RNAi (VDRC #6299), *GMR-*Gal4 (BDSC #8440), *ninaE-*Gal4 (BDSC #1104), *repo-*Gal4 (BDSC #7415), *pdf*-Gal4 (BDSC #6900), *Eaat1*-Gal4 (BDSC #8849), *Lama*-Gal4 (BDSC #35543), *per^0^* (Smith and Konopka, 1981), *yw*;P{EPgy2}*bel^EY 08943^* (BDSC #19945); P{PZ}*bel^cap-1^ry^506^*/TM3, *ry^RK^ Sb^1^ Ser^1^* (BDSC #11778), *bel::GFP* (Morin et al., 2001) (Kyoto Stock Centre #*ZCL2696*), *UAS-CD8-GFP* (BDSC #5137). Co-IP was performed with *yw; tim-GAL4/+; UAS-HAcry/+* (Dissel et al., 2004). Flies were maintained on a standard cornmeal medium under LD 12:12 regime and at constant 23°C.

### Co-immunoprecipitation, Mass Spectrometry Analysis, and Western Blot

Head extracts from overexpressing HACRY flies raised in 12:12 light:dark cycles and collected at Zeitgeber Time 24 (ZT24), before lights on, and after a 15-min light pulse were subjected to coimmunoprecipitation and Mass Spectrometry analysis as previously described (Mazzotta et al., 2013). Immunocomplexes were analyzed by Western blot using the following antibodies: rabbit polyclonal anti-BELLE (1:2000) (Johnstone et al., 2005) and mouse anti-HA (1:5000; Sigma Aldrich, St. Louis, MO, United States). Anti-rabbit IgG HRP (1:3000; BioRad Laboratories, Hercules, CA, United States) and anti-mouse IgG HRP (1:5000; Sigma Aldrich, St. Louis, MO, United States) were used as secondary antibodies.

### RNA Extraction and qRT-PCR

RNA was extracted using the reagent Trizol (Invitrogen) as indicated in manufacturer’s instructions. For the analysis of *belle* expression, around 50 heads from flies reared in LD cycles for at least 3 days and collected at the indicated time points were used. For the analysis of transposons 30 mg of male or female gonadal tissues or heads were used. 1 μg of total RNA was used as a template for oligonucleotide dT-primed reverse transcription using SuperScriptIII RNaseH-reverse transcriptase (Invitrogen), according to manufacturer’s instructions. qRT-PCR was performed in the 7500 Real-TimePCR System (Applied Biosystem) using SYBR green (Celbio) according to the manufacturer’s instruction. For quantification of the transcripts, we used the 2ΔΔCt method as previously reported (Specchia et al., 2010). The primers used are listed in Supplementary Table S1.

### Immunocytochemistry

For the analysis of BELLE localization, heads from 7 days old males were fixed in 4% paraformaldehyde in phosphate buffer saline (PBS; pH 7.4) for 4 h, then they were washed in PBS twice and cryoprotected by incubation in 12.5% sucrose for 10 min and in 25% sucrose at 4°C overnight. Material was embedded in Tissue-Tek, frozen in liquid nitrogen, and cryostat 20 μm sections were cut. The sections were washed in PBS for 30 min and five times in phosphate buffer (PB) with an addition of 0.2% Triton X-100 (PBT). After that, sections were incubated in 5% normal goat serum (NGS) with an addition of 0.5% bovine serum albumin (BSA) for 30 min first at room temperature, and then incubated with primary antibodies anti-REPO (mouse, 1:500, Hybridoma Bank), anti-Ebony (rabbit, 1:750, a gift from Dr. B. T. Hovemann, Ruhr Universität Bochum) and anti-GFP (rabbit, 1:1000, Novus Biologicals) for 24 h. Afterward, sections were washed six times in PBT/BSA, and blocked in 5% NGS for 45 min. After that, goat anti-mouse (conjugated with Cy3) and goat anti-rabbit (conjugated with Alexa 488) secondary antibodies (both 1:500, Jackson Immuno Research) were applied overnight in 4°C. Finally, sections were washed twice in BSA, six times in PBT, and twice in PBS. Then, cryosections were mounted in Vectashield medium (Vector) and examined with a Zeiss Meta 510 Laser Scanning Microscope. About 30 brains or cryosections were checked.

For the analysis of PER in clock-neurons, flies were synchronized by LD cycles at least for 3 days and then collected under LD or DD conditions at the indicated time points. Whole flies were quickly fixed for 2 h in PFA 4%. About 10–12 brains were dissected in PBS and then treated as previously described (Vanin et al., 2012). Average fluorescence values are reported.

### Male and Female Fertility Analysis

One young male was mated to three control virgin females, and three virgin females were mated with three control males. Ten individual males and 30 females were tested for each genotype. After 4 days, the crosses were transferred to a fresh vial. The parental flies were removed from the last vial after an additional 4 days. The number of the adult progeny from each vial was counted.

### Behavioral Analysis

Locomotor activity of individual flies was recorded for 3 days in LD and 7 days in DD using the Drosophila Activity Monitoring System (TriKinetics, Inc., Waltham, MA, United States). Data were collected every 5 min and then analyzed in 30 min bins using spectral analysis and autocorrelation (Zordan et al., 2007). Morning anticipations was detected fly-by-fly examining the activity mean of 3 days under LD conditions (Vanin et al., 2012) and the Morning Index was calculated as in Seluzicki et al. (2014).

### Statistical Analyses

For comparisons between two measurements a two-tailed Student’s *t*-test was used to show the significance level of the replicated experiments. Expression profile’s significance was evaluated by non-parametric ANOVA, Kruskal–Wallis test (*p*-value < 0.05) whereas putative period and phase of oscillation were identified by RAIN non-parametric test for independent samples (adjusted *p*-value < 0.05) (Thaben and Westermark, 2014).

## Results

### dCRY Interacts With the RNA Helicase BELLE

In an experiment aimed at the identification of new genes involved in the circadian machinery in *Drosophila*, a coimmunoprecipitation assay followed by mass spectrometry analysis was performed on transgenic flies overexpressing a hemagglutinin (HA)-tagged form of dCRY (HACRY) raised in 12:12 light:dark cycles and collected at Zeitgeber Time 24 (ZT24), before lights on, and after a 15-min light pulse (Mazzotta et al., 2013).

**FIGURE 1:**
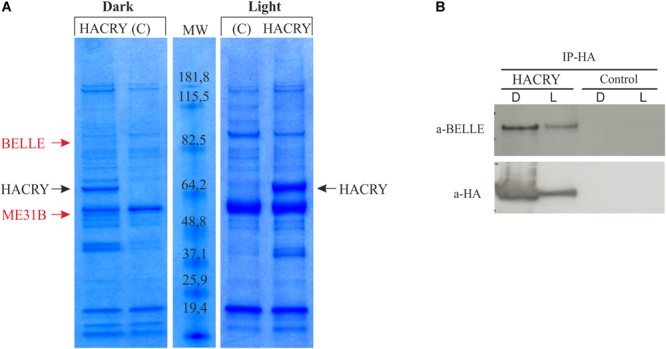
dCRY interacts with BELLE. **(A)** Coomassie blue-stained gel of heads protein extracts coimmunoprecipitated with an anti-HA antibody. HACRY-overexpressing flies (HACRY, *yw;tim-GAL4/+; UASHAcry/+*) and relative controls (C, *yw;tim-GAL4*) were reared in 12:12 light: dark and collected in the dark (ZT24) and in the light (ZT24 + 15-min light pulse). Molecular masses of markers are indicated (BenchMark Pre-Stained Protein Ladder; Invitrogen). MW, molecular weight. Bands corresponding to HACRY are indicated in black, while stained proteins excised and characterized by mass spectrometry are indicated in red. **(B)** Co-immunoprecipitation and Western blot confirming the interaction between HACRY and BELLE in HACRY-overexpressing flies (HACRY, *yw;tim-GAL4/+; UASHAcry/+*). *tim-GAL4* flies were used as control. Heads were collected as in **(A)**. Membranes were probed with anti-BELLE and anti-HA antibodies.

A ∼85-kDa species was observed in the sample in the dark that was not present in the negative control (Figure 1A). This protein band was digested in-gel, and the peptide mixtures analyzed by liquid chromatography–mass spectrometry (LC-MS)/MS using an ESI-QTOF mass spectrometer (Wilm et al., 1996). Analysis of the MS/MS data using the MASCOT software yielded the identification of BELLE (Supplementary Table S2), a DEAD box RNA helicase involved in RNA metabolism at several levels (Linder and Jankowsky, 2011).

The presence of BELLE in the complex with HACRY was also confirmed by Western blot with an anti-BELLE antibody (Johnstone et al., 2005). By this procedure, BELLE was also detected after a 15 min of light pulse (Figure 1B). The same blot was hybridized also with an anti-HA antibody, in order to assess the specificity of the interaction (Figure 1B). The signal corresponding to BELLE was more intense in head collected in the dark; in fact after 15 min of light pulse the amount of BELLE was lower, in line with that of HACRY that decreased due to the well-known light-dependent degradation of CRY (Peschel et al., 2009).

### The RNA Helicase BELLE Is Expressed in the Fly Brain

It is known that BELLE is expressed in germ cells throughout all developmental stages and in larval neurons (Johnstone et al., 2005; Rolls et al., 2007), but little information was available regarding its expression in the adult fly head.

**FIGURE 2:**
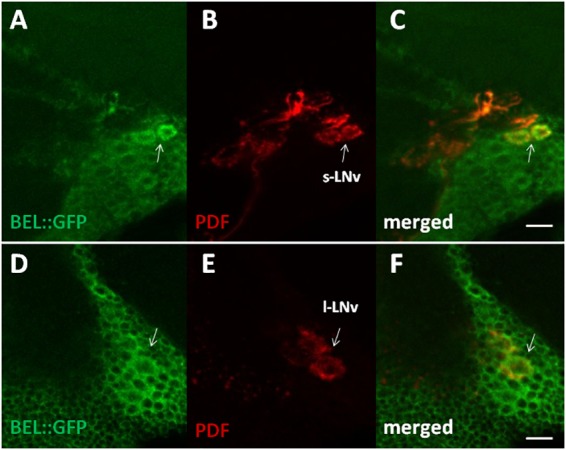
BELLE localizes in the main pacemaker cells in the brain of *Drosophila melanogaster*. Confocal images of whole mount of adult brain expressing BELLE fused with green fluorescent protein (*bel::GFP*). Scale bar: 10 μm. **(A,D)** Anti-GFP immunostaining (BEL::GFP). **(B,E)** Anti-PDF was used to label PDF-expressing ventral lateral neurons. Depends on optical section small LNv (s-LNv) or large LNv (l-LNv) are visualized. **(C,F)** BEL::GFP and PDF immune staining co-localize in the area of LNv cell bodies.

**FIGURE 3:**
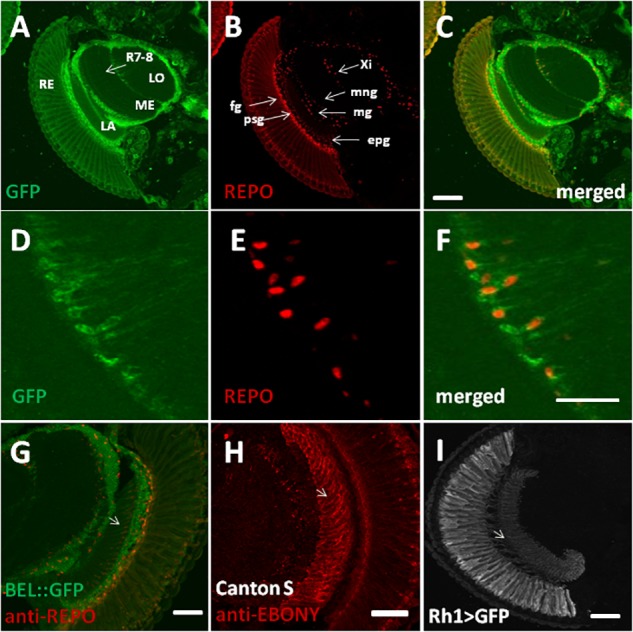
BELLE localizes in the visual system of *Drosophila melanogaster*. Confocal images of horizontal cryosection of adult brain expressing BELLE fused with green fluorescent protein (*bel::GFP*). Anti-REPO was used to label all glia nuclei. **(A)** BEL::GFP signal is localized in the optic neuropils: lamina (LA), medulla (ME), and lobula (LO). Weak signal is visible in R7 and R8 photoreceptor terminals (R7-8) in the medulla. Signal in the retina (RE) is probably due to autofluorescence. **(B)** Anti-REPO staining allowed recognized different glia types: fenestrated glia (fg), pseudocartridge glia (psg), epithelial glia (epg), medulla glia (mg), marginal glia (mng), and inner chiasm glia (Xi). **(C)** Merged signal showed localization of BELLE in glial cells. Bar 50 μm. **(D–F)** Higher magnification of inner chiasm glia showed that BELLE is localized mostly in the cytoplasm. Bar 20 μm. **(G–I)** Localization of BELLE in the first optic neuropile, called lamina, analyzed on confocal images of horizontal cryosection of adult brain. Bar: 50 μm. **(G)** Anti-GFP and Anti-REPO immunostaining showing that BEL::GFP signal is higher in the distal lamina (close to retina), where interneurons cell bodies are located. Weak labeling is also visible across the lamina, forming regular columns. Glia nuclei staining overlaps with observed BELLE location, suggesting that this signal originates from epithelial glia. **(H)** Section of wild-type *CantonS* brain stained with anti-EBONY to label epithelial glial cells in the lamina and their specific columnar shape (see Richard et al., 2002). **(I)** Anti-GFP staining on section of Rh1 > GFP brains showing R1–R6 photoreceptors terminals in the lamina.

In order to characterize BELLE expression in the fly brain, we performed an immunocytochemistry experiment on flies carrying a GFP exon trap in *belle* (*bel::GFP*) (Morin et al., 2001). Flies were maintained in standard light–dark conditions, brains were dissected and an anti-GFP antibody was used to detect BEL::GFP expression. We observed BELLE expression in clock neurons and in glial cells in the optic lobe, with a predominantly cytoplasmic localization (Figure 2, 3). In clock neurons, BELLE was expressed in either the small or the large ventral lateral neurons (s-LNvs and l-LNvs respectively) (Figure 2), PDF-expressing cells that act in promoting and governing the circadian locomotor behavior. In glia we observed BELLE expression in the epithelial glia and a weak labeling also in the lamina (Figure 3D–F): position and shape of these GFP- labeled cells suggest they may be R1-R6 photoreceptor terminals (Figure 3D–F); however, anti-REPO staining showed glia cells nuclei at the top of each structure (Figure 3G). Observed columnar shape is also similar to anti-EBONY staining (Figure 3H), which is a marker for epithelial glia (Richard et al., 2002), suggesting that BELLE may be located in epithelial glia in the lamina. Moreover, we observed immunostaining for BEL::GFP also in giant inner chiasm glia, which connects the medulla with the lobula complex in the optic lobe (Kremer et al., 2017). In these cells, both BELLE and PER are expressed (Supplementary Figure S1).

### BELLE Expression Shows Circadian Features

In order to characterize the temporal expression of *belle*, mRNA and protein levels were analyzed during the 24 h. Wild-type flies *white^1118^* were synchronized by 12:12 LD cycles and collected every 3 h, either in LD regime or at the third day after being transferred to constant darkness (DD).

**FIGURE 4:**
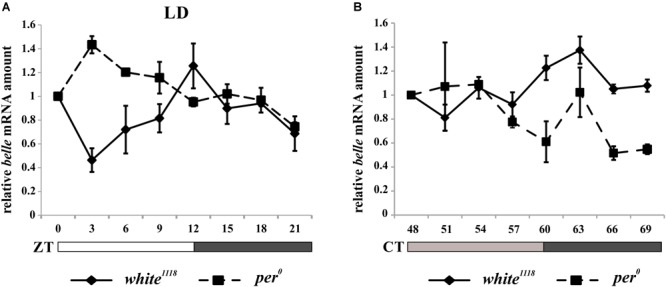
*belle* mRNA expression shows circadian features. qRT-PCR on heads from *white^1118^* and *per^0^* flies reared in 12.12 LD **(A)** or over the third day in DD **(B)**. Relative abundance of *belle* mRNA was defined as a ratio with *rp49*. Values represent mean levels ± SEM of three biological replicates. The value corresponding to CT 54 in *per^0^* flies represents mean level of two biological replicates.

*belle* expression appeared to oscillate both in LD and in DD conditions. In particular, in LD cycles the level of differential expression during the 24 h was just below the limit of significance (Kruskal–Wallis *p*-value 0.06); nevertheless, the algorithm RAIN suggested the presence of a rhythmic expression pattern (adjusted *p*-value 2,61E-02) with a peak at ZT12 (Figure 4A). In constant darkness *belle* mRNA showed differential expression around the 24 h (Kruskal–Wallis *p*-value 0.03), and a peak at CT63 was identified (RAIN *p*-value 1,92E-05) (Figure 4B). These results are in line with a previous microarray study showing a peak of expression for *belle* at ZT17 (Claridge-Chang et al., 2001). In *per*^0^ mutant, *belle* oscillation is maintained in LD cycles, although with a peak at ZT3 (Kruskal–Wallis *p*-value 0.02, RAIN *p*-value 4,91E-07) (Figure 4A), while it was lost in constant conditions (Kruskal–Wallis *p*-value 0.609) (Figure 4B).

The analysis of BELLE in fly heads by western blot showed no significant protein oscillation, neither in LD nor in DD (Kruskal–Wallis *p*-values 0.9 and 0.8, respectively) (Supplementary Information and Supplementary Figure S2). This result is in line with that obtained by co-immunoprecipitation (Figure 1B): the lower amount of BELLE pulled down in light compared to dark uniquely depends on the light-mediated degradation of CRY.

### BELLE Affects Circadian Locomotor Activity

In order to unravel a possible involvement of *belle* in the generation of circadian rhythmicity in *Drosophila*, we depleted the expression of the gene by RNAi in both glia and clock neurons.

When we knocked down *belle* with the pan-glial driver *repo-*Gal4 (Awasaki and Ito, 2004) and *lama*-Gal4, a driver for glial precursor cells, lamina precursor cells and lamina neurons (Apitz and Salecker, 2016), we observed developmental lethality. The same result was obtained by KD in all canonical clock neurons and clock relevant glial cells expressing *timeless* (with a *tim-*Gal4) (Emery et al., 1998). This result is in agreement to what reported for the null allele of *belle* (Johnstone et al., 2005). When we depleted *belle* in PDF expressing neurons and a restricted subset of glial cells (with *pdf*-Gal4 and *Eaat1*-Gal4, respectively) (Renn et al., 1999; Rival et al., 2004), flies were viable and endogenous rhythmicity was not altered (Table 1); only a slight impairment in the morning anticipation of locomotor activity in LD was observed (Table 2). In contrast, RNAi against *belle* in the photoreceptor cells (*GMR-*Gal4 and *ninaE-*Gal4) did not affect either vitality or behavior (Supplementary Table S3).

**Table 1:** Locomotor activity of *belle* Knock-Down flies under constant darkness (DD).

Genotype	N Tot	N Alive	N R	% R	τ		*SEM*
*w;pdf-Gal4/+;UAS-RNAi-belle/+*	63	55	48	87.27	24.95	±	0.12
*w;Eaat-Gal4/+;UAS-RNAi-belle/+*	32	31	27	87.10	24.01	±	0.07
*w;pdf-Gal4/+*	30	27	26	96.30	24.49	±	0.09
*w;Eaat-Gal4/+*	30	29	26	89.66	24.08	±	0.06
*w;;UAS-RNAi-belle/+*	47	39	33	84.62	23.96	±	0.07

*R: rhythmic flies. The experiments were performed at 23°C.*

**Table 2:** Locomotor activity of *belle* Knock-Down flies in entrainment conditions (LD).

Genotype	MA (%)	EA (%)	MI		*SEM*	*N*
*w;pdf-Gal4/+;UAS-RNAi-belle/+*	54.55	92.73	0.069^a^	±	0.025	55
*w;Eaat-Gal4/+;UAS-RNAi-belle/+*	67.74	90.32	0.274	±	0.034	31
*w;pdf-Gal4/+*	74.05	100	0.141	±	0.038	27
*w;Eaat-Gal4/+*	86.21	100	0.254	±	0.038	29
*w;;UAS-RNAi-belle/+*	82.05	100	0.109	±	0.022	39

*t-Test was performed. ^*a*^: ns vs. *w;pdf-Gal4/+* and *w;;UAS-RNAi-belle/+.* MA: morning anticipation; EA: evening anticipation. MA and EA were detected, fly-by-fly, examining the bout of activity prior to light transitions. MI: Morning Index. The experiments were performed at 23°C.*

**FIGURE 5:**
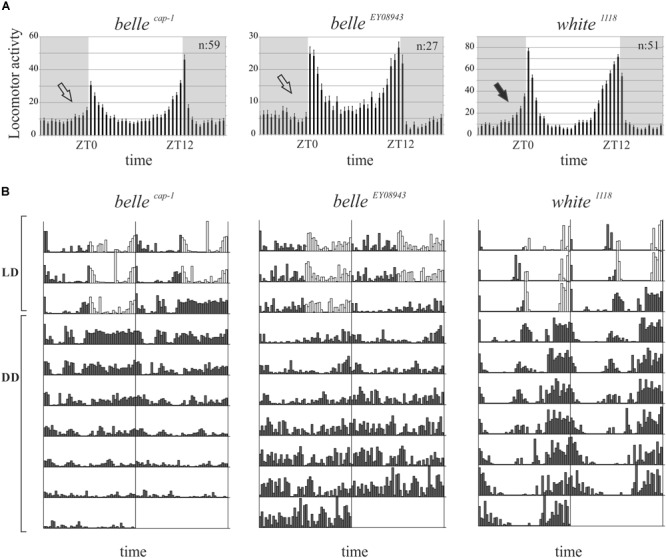
BELLE affects circadian locomotor activity. **(A)** Activity means of flies over 3 days of LD cycles ± SEM. N: number of flies; gray boxes: dark phase. *Belle* mutants lost the morning anticipation (empty arrows) compared to control (black arrow). ZT0: lights-on, ZT12: lights-off. **(B)** Representative double-plotted locomotor activity of a single fly recorded for 3 days under LD and 7 days under DD conditions (gray bars: dark phase; white bars: light phase). LD: light–dark cycles; DD: constant darkness.

We then decided to investigate a possible role for *belle* in the circadian machinery using two viable mutant lines generated by p-element insertion in the regulatory region (*belle ^cap-1^* and *belle ^EY 08943^*) (Bellen et al., 2004). Flies were entrained for 3 days in light dark cycles at constant temperature (23°) followed by 7 days of constant darkness. Either mutant exhibited an impairment of the locomotor behavior (Figure 5): in LD conditions, both lines presented the canonical bimodal profile, but a severe loss of the morning anticipation of the locomotor activity was displayed (Figure 5A and Table 3). A high percentage of flies showed also an arrhythmic behavior in DD, although no defects were monitored concerning the period, which was comparable to wild-type (Figure 5B and Table 4).

**Table 3:** Locomotor activity of *belle* mutants in entrainment conditions (LD).

Genotype	MA (%)	EA (%)	MI		*SEM*	*N*
*belle ^cap-1^* ^a^	42.37	81.36	0.022^a^	±	0.033	59
*belle ^EY 08943^* ^b^	14.81	70.37	0.026^b^	±	0.041	27
*white ^1118^*	94.12	98.04	0.179	±	0.024	51

**Table 4:** Locomotor activity of *belle* mutants in constant darkness (DD).

Genotype	N Tot	N Alive	N R	% R	τ		*SEM*
*belle ^cap-1^* ^a^	85	59	40	67.80	24.24	±	0.10
*belle ^EY 08943^* ^b^	46	27	8	29.63	24.36	±	0.25
*white ^1118^*	54	51	45	88.24	24.44	±	0.12

*R: rhythmic flies. The experiments were performed at 23°C.*

### Altered PER Cycling in Circadian Pacemaker Neurons in Belle Mutants

We performed PER staining on brains dissected every 4 h from flies collected under LD cycles and during the 5th day of DD conditions, in order to assess whether the impairment of the circadian locomotor activity of the *belle* mutants could be due to an altered PER cycling in the pacemaker neurons. PDF expressing cells were identified by co-staining for this peptide.

**FIGURE 6:**
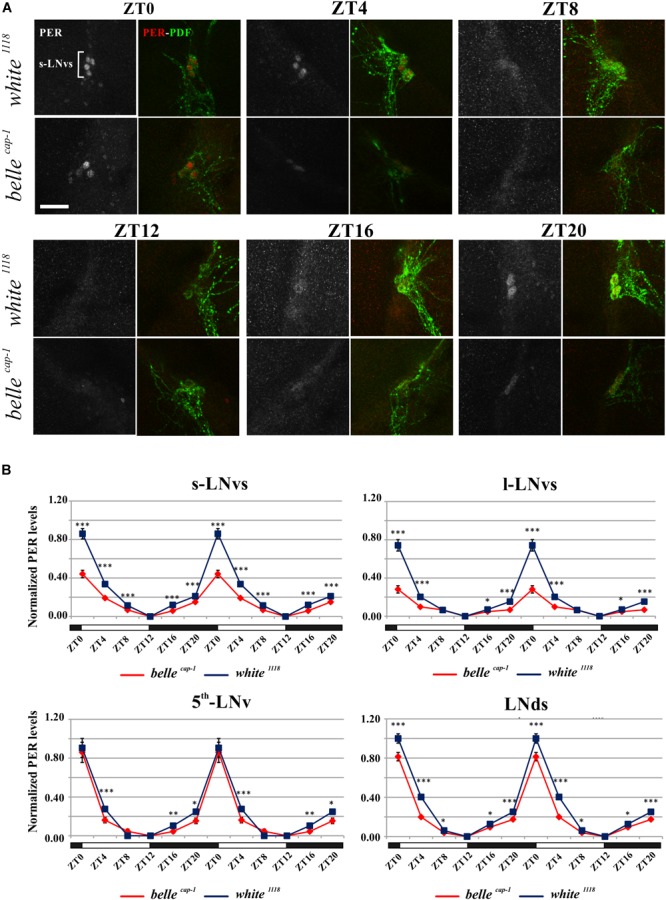
Altered PER expression in *belle ^cap-1^* mutant in entrainment conditions. PER oscillations in the clock Lateral Neurons of *belle ^cap-1^* mutant flies in LD cycles. Flies were collected at the indicated time points after 3 days in LD and stained for PER and PDF. **(A)** Representative confocal stack images of PER staining in s-LNvs. *belle ^cap-1^* mutants displayed lower PER expression in circadian pacemaker neurons, compared to control (*white ^1118^*). Scale bar: 25 μm. **(B)** PER quantification in clock Lateral Neurons. Peak value was set to 1 and the rest of the values were normalized accordingly. Data are shown as double-plotted. *t*-Test: ^∗∗∗^*p* < 0.005, ^∗∗^*p* < 0.01, ^∗^*p* < 0.05.

**FIGURE 7:**
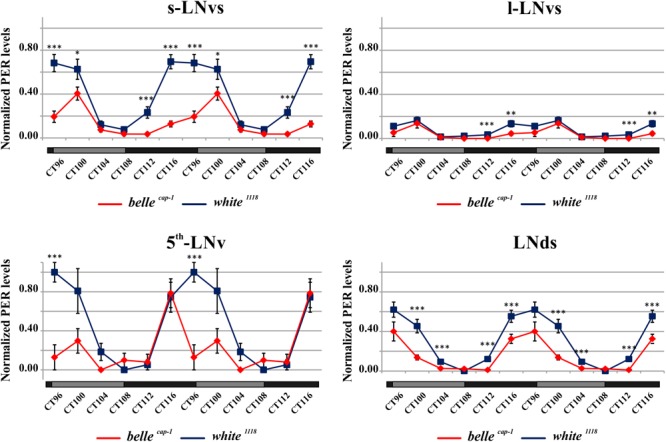
Altered PER expression in *belle ^cap-1^* mutant in constant conditions. PER quantifications in the clock Lateral Neurons of *belle ^cap-1^* mutant flies in constant darkness. Flies were first entrained for 2 days in LD and then analyzed after 4 days of DD. Only arrhythmic flies were collected every 4 h and stained for PER expression during the 5th day of DD. Peak value was set to 1 and the rest of the values were normalized accordingly. Data are shown as double-plotted. *t*-Test: ^∗∗∗^*p* < 0.005, ^∗∗^*p* < 0.01, ^∗^*p* < 0.05.

Mutant lines showed defects in some clusters of neurons. Under LD conditions, *belle ^cap-1^* flies exhibited a significant reduction of oscillation amplitude in both s-LNvs and l-LNvs, particularly marked at ZT0 (Figure 6). Similarly, in constant darkness, a general decrease of PER staining was observed compared to wild-type, which was particularly enhanced during the subjective night/beginning of the subjective day (Figure 7). Quantification of PER levels indicated that the greatest effect occurs in the small PDF cells (s-LNvs) (Figure 7), those cells that normally show high PER expression and also govern the behavioral phenotype in constant conditions (Grima et al., 2004). Besides a decrease of its levels, in these cells the accumulation of PER is slow down, with a peak delayed of about 4 h with respect to control (Figure 7). In large PDF neurons (l-LNvs) PER cycling is dampened, similarly to control and in accordance to previous observations (Grima et al., 2004).

The *bel^EY 08943^* mutant exhibited a less striking phenotype: a significant difference was observed in the PDF positive s-LNvs only in constant darkness, where the oscillation of PER was 4 h delayed compared to control (Supplementary Figure S3), similarly to what described in *bel^cap-1^* flies (see above).

Interestingly, both *belle* mutants were characterized by a reduction in the number of l-LNvs: a high percentage of brains, in fact, presented three neurons instead of the canonical four (83.93, 76.28, and 6.78% in *bel ^cap-1^, bel ^EY 08943^*, and *white ^1118^*, respectively).

### BELLE Influences the Expression of Specific TEs in the Fly Heads and Gonads

As BELLE co-localizes with its paralog Vasa, a key component of the piRNA pathway-mediated regulation of the TEs, in ovaries and testes (Johnstone et al., 2005), we decided to further investigate about a possible involvement of *belle* in the piRNA-mediated regulation of TEs elements.

**FIGURE 8:**
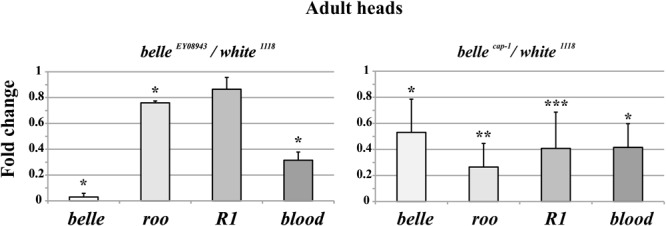
Altered TEs expression in *belle* mutant heads. qRT-PCR on heads from *belle ^EY 08943^, belle ^cap-^*^1^, and *white ^1118^* flies reared in 12:12 LD cycles and collected at ZT3. Represented are relative amount of *belle* and three transposable elements (TEs) (*roo, R1*, and *blood*) in *belle ^EY 08943^* **(A)** and in *belle ^cap-^*^1^ **(B)** compared to *white ^1118^* control, to which an arbitrary value of 1 was assigned. Represented are mean ± SD of three biological replicates. ^∗∗∗^*p* < 0.005, ^∗∗^*p* < 0.01, ^∗^*p* < 0.05.

We analyzed the RNA expression of specific transposons in heads of adult *belle ^EY 08943^* and *belle ^cap1^* flies. In *belle ^EY 08943^* two of them, *roo* and *blood*, showed a significant reduction in their expression compared to control (Figure 8, left panel). In addition, *R1* also exhibited a slight reduction in the RNA amount, though not significant. In *belle ^cap-^*^1^ the TE transcripts were greatly reduced, also compared to *belle ^EY 08943^*, and even *R1* exhibited a significant reduction compared to control (Figure 8, right panel). These results are in disagreement with other piRNA mutants, that normally show an increased amount of the TE transcripts (Vagin et al., 2006; Brennecke et al., 2007; Ghildiyal and Zamore, 2009; Khurana and Theurkauf, 2010; Specchia et al., 2010; Piacentini et al., 2014; Bozzetti et al., 2015). Nevertheless, based on our observations, a role for *belle* in the regulation of the TEs expression can still be hypothesized.

In order to better elucidate this involvement, we decided to investigate *belle* function in the gonads, where the role of the different components of the piRNA pathway is almost entirely known (Vagin et al., 2006; Brennecke et al., 2007; Klattenhoff and Theurkauf, 2008; Li et al., 2009; Malone et al., 2009; Khurana and Theurkauf, 2010; Senti and Brennecke, 2010; Czech et al., 2013; Handler et al., 2013; Muerdter et al., 2013; Iwasaki et al., 2015). Our group has identified some key components of the piRNA pathway, like *aubergine, hsp83, dFmr1* (Specchia et al., 2008, 2010, 2017; Specchia and Bozzetti, 2009; Bozzetti et al., 2015) and demonstrated that TEs and repetitive sequences like *Stellate* are transcriptionally activated as a consequence of mutations in these piRNA genes (Aravin et al., 2001, 2003; Nishida et al., 2007; Specchia and Bozzetti, 2009; Bozzetti et al., 2012, 2015; Malone et al., 2015; Sahin et al., 2016).

**FIGURE 9:**
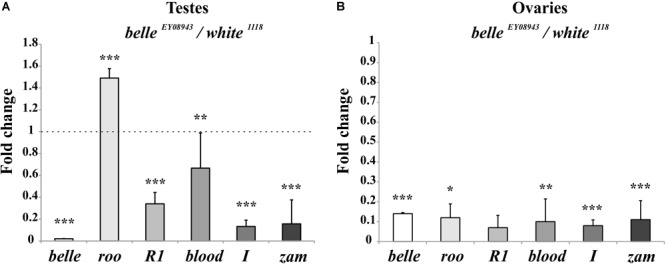
Altered TEs expression in *belle* mutant gonads. qRT-PCR on ovaries **(A)** and testes **(B)** from *belle ^EY 08943^* and *white ^1118^* flies reared in 12:12 LD cycles and collected at ZT3. Represented relative mRNA levels of *belle* and five TEs (*roo, R1, blood, I-element*, and *ZAM*) in *belle ^EY 08943^* compared to *white ^1118^*, control, to which an arbitrary value of 1 was assigned. Represented are mean ± SD of three biological replicates. ^∗∗∗^*p* < 0.005, ^∗∗^*p* < 0.01, ^∗^*p* < 0.05.

We first tested the expression of some transposons in gonads: a dramatic reduction of transposons transcript was observed in ovaries from *belle* mutants in comparison to wild-type (Figure 9B and Supplementary Figure S4), similarly to what observed in heads. A similar reduction was also observed in *belle ^EY 08943^* testes, with the exception of *roo*, whose transcript increases in *belle ^EY 08943^* compared to control (Figure 9A).

We have also performed a fertility test, showing that mutant males are completely sterile with respect to controls (average progeny of 128), while females exhibit a severe reduction of fertility (average progeny of 6,6 in mutants with respect to 43,4 in control) (Supplementary Figure S5). This observation is in agreement to what reported for other alleles of *belle* (Johnstone et al., 2005).

## Discussion

### BELLE Is a Putative Circadian Clock Component

Aiming at finding new molecular component of the clock machinery in *Drosophila*, we have identified the DEAD-box RNA helicase BELLE as interactor of CRY. DEAD (Asp-Glu-Ala-Asp)-box RNA helicases are highly conserved proteins known to play central roles in essentially every stages of RNA metabolism, in both nucleus and cytoplasm (Linder and Fuller-Pace, 2013). BELLE has been implicated in splicing and translational regulation (Worringer et al., 2009; Ihry et al., 2012) and, more recently, in miRNA and siRNA pathways (Pek and Kai, 2011). It is essential for viability, organismal growth, and fertility (Johnstone et al., 2005).

The involvement of a RNA helicase in the circadian clock is not uncommon. In *Neurospora crassa*, in fact, two members of DEAD-box RNA helicase family play important roles in the circadian machinery: FRH (Frequency-interacting RNA Helicase), that regulates the stability of FREQUENCY (FRQ), thus acting on both negative and positive circadian feedback (Cheng et al., 2005; Guo et al., 2010; Shi et al., 2010; Lauinger et al., 2014), and PRD-1 (PERIOD-1), ortholog of human DDX5 and DDX17, specifically regulating circadian rhythmicity also in FRQ-less oscillators (Emerson et al., 2015; Adhvaryu et al., 2016).

BELLE has been extensively studied for its crucial role in the *Drosophila* gonads, where it is essential for viability and fertility (Johnstone et al., 2005; Kibanov et al., 2011). However the presence of BELLE has been reported also in *Drosophila* neurons, specifically in the neuronal cell body (Beckham et al., 2008), and proteomic data describe its expression in adult fly heads (Aradska et al., 2015).

We have depicted BELLE expression in both PDF expressing clock neurons and in the glial cells in the optic lobe. It is reported that adult glial cells contain a molecular clock and evidences about their role in the modulation of circadian rhythmicity are continuously accumulating (Siwicki et al., 1988; Zerr et al., 1990; Ewer et al., 1992; Pyza and Gorska-Andrzejak, 2004; Suh and Jackson, 2007; Damulewicz et al., 2013; Ng and Jackson, 2015; Ng et al., 2016). Nevertheless, the signaling between glia and the clock neurons that control the locomotor behavior is not yet fully understood. Many PER-expressing glial cells, such as epithelial glial, are located in proximity of clock neurons or their processes (Suh and Jackson, 2007), suggesting a direct communication. Epithelial glia affects rhythmic behavior also by regulating neurotransmitter metabolism through Ebony (Suh and Jackson, 2007). BELLE is mostly localized in the cytoplasm: this observation is in accordance with previous reports of a predominantly cytoplasmic distribution for BELLE in fly gonads (Johnstone et al., 2005) and its localization in cytoplasmic processing bodies (P-bodies), where it is supposed to act as translational repressor (Beckham et al., 2008). At the transcriptional level, we observed a clear daily rhythm for *belle* mRNA either in LD and DD; the phase of the oscillation is altered in *per*^0^ flies in LD, while it is abolished in constant conditions, suggesting that the circadian clock controls the expression of this helicase.

These results are perfectly in line with previously reported observations: in fact, a microarray study showed a peak of expression for *belle* at ZT17 (Claridge-Chang et al., 2001) and, in a chromatin immunoprecipitation (ChIP) analysis, *belle* was found to be a direct target of CLK in fly heads (Abruzzi et al., 2011). The peak-time of *belle* mRNA in the late day/early night is in agreement with a direct control of CLOCK in the expression of this gene, since it is reported that CLK-CYC bind to E-box of their target genes in the late day (reviewed in Peschel and Helfrich-Forster, 2011).

The effect of down-regulation of *belle* in glia and in TIM-expressing cells, lethality and arrested growth at larval stage, respectively, is in agreement with previously reported observation (Johnstone et al., 2005). In fact, strong alleles of *belle* are zygotic lethal and larvae arrest their development at stage L1, which is prolonged up to 5 days (Johnstone et al., 2005). This larval growth arrest phenotype is common to other genes encoding for translation factors, such as elongation factors *elF4A* and *elF4E* (Galloni and Edgar, 1999; Lachance et al., 2002), for which a lethality was observed also when knocked down in TIM cells (Bradley et al., 2012). The down-regulation of *belle* in a restricted subset of clock neurons or glial cells resulted in the loss of the morning anticipation in about 60% of flies reared in entrainment conditions, suggesting a possible role for BELLE in the regulation of morning activity. This phenotype was much stronger in two viable allele of *belle*: in LD cycles, in fact, a high proportion of flies were devoid of morning anticipatory, further indicating that the small ventral lateral neurons, that constitute the morning oscillator (Grima et al., 2004), are affected in these flies. In constant conditions the percentage of rhythmic flies in the two *belle* mutants was significantly lower compared to wild-type, reinforcing the fact that the activity of lateral ventral neurons, that drive also the endogenous behavioral rhythmicity (Helfrich-Forster, 1998; Renn et al., 1999; Lin et al., 2004), are impaired by *belle* mutation.

We sought to analyze PER cycling in clock neurons of *belle* mutants, in order to determine whether an altered PER expression could account for the behavioral defects of these flies. In fact PER oscillation in circadian pacemaker neurons is a hallmark of a functioning molecular oscillator, and an impairment of LNvs activity results in a de-synchronization of PER cycling within different groups of circadian neurons (Renn et al., 1999; Peng et al., 2003; Lin et al., 2004).

Although with a different magnitude between the two mutants, a general decrease of PER staining was observed compared to control. In entrainment conditions, in *belle ^cap-1^* flies the amplitude of PER oscillation was significantly reduced in both s-LNvs and l-LNvs, and the difference was particularly marked at ZT0. In constant conditions, s-LNvs displayed the stronger effect, with a PER reduction more pronounced at the end of subjected night/beginning of subjective day, when the protein is expressed at maximum levels. Moreover, in these cells the accumulation of PER was also slow down, reaching maximum levels about 4 h later than the control. The *belle ^EY 08943^* mutant exhibited a less striking phenotype: only in constant darkness PDF positive s-LNvs displayed a 4 h delay compared to control. Altered PER oscillation in PDF-positive LNvs may account for the defects in the morning anticipation of the locomotor activity of *belle* mutants. These flies did not display particular defects in PDF projections/arborizations, but a reduction in the number of large ventral lateral neurons (l-LNvs) was observed. The altered PER cycling in clock neurons is reminiscent of the phenotype observed in mutants for RNA binding proteins involved at different levels in the post-transcriptional regulation of circadian clock, such as the translational factors NAT1 (Bradley et al., 2012) and ATAXIN2 (ATX2) (Lim and Allada, 2013; Zhang et al., 2013). ATX-2 is a RNA-binding protein that very recently has been shown to sustain circadian behavior in *Drosophila* functioning as both activator and repressor of translation, and this switch is mediated by complex formation with two specific factors, LSM12 and ME31B/DDX6, respectively (Lim and Allada, 2013). *Lsm12* mutant flies exhibited a dampened PER cycling in circadian pacemaker neurons, that resulted in a lengthening of the locomotor activity period (Lee et al., 2017). ME31B is the homolog of human DDX6, a RNA helicase belonging to the same family of DEAD-box ATP-dependent helicase of BELLE, and flies in which the expression of this gene was depleted exhibit very poor rhythmicity (Lee et al., 2017). All these similarities led us to hypothesize that also BELLE could play a role in the post-transcriptional control of the circadian clock in *Drosophila*, probably in mechanisms in which the aforementioned factors are also involved. This idea is supported by the fact that we have found ME31B in our Co-IP and MS analysis in the same complex as BELLE in fly heads (Figure 1 and Supplementary Table S2), and that the two proteins belongs to the same translational repression complex that in *Drosophila* embryo is responsible for both repression and degradation of non-localized *nanos* mRNA, in a mechanism that involves also the Piwi-interacting RNA (piRNA) machinery (Gotze et al., 2017).

### BELLE Has a Role in the Regulation of the TEs in the Nervous System and in Gonads

BELLE has a well-studied role in the *Drosophila* gonads (Johnstone et al., 2005; Kibanov et al., 2011; Kotov et al., 2016). In this study we have observed that loss of *belle* function impinges on the expression of TEs either in the brain or in the gonads, with an impact on the fertility of both males and females. We have observed a reduction of the transposons’ transcription in *belle* mutant, in comparison to wild-type; notably, other piRNA mutants normally exhibit an increased TEs’ expression (Aravin et al., 2007; Specchia et al., 2008, 2017; Specchia and Bozzetti, 2009; Senti and Brennecke, 2010). This observation well-agrees with the recently reported data on the effect of *belle* mutants in the silencing of P-transposon-derived constructs in ovaries, depending on *de novo* piRNAs generated from the transgenes in the region of their insertion (Lo et al., 2016). Our data led us to infer that *belle* regulates the transposons probably *via* the piRNA-mediated pathway. This hypothesis is somehow supported by the fact that Vasa and Spindle-E, two members of the same DEAD box protein family, have a well-recognized role in the piRNA pathway in ovaries and testes, though their loss of function results in an up-regulation of TEs (Lasko, 2013; Iwasaki et al., 2015; Specchia et al., 2017), as opposite to that of *belle*. They also co-localize at the “nuage” of the germline where, Argonaute, Tudor and many other factors are also located for an efficient TE silencing (Lasko and Ashburner, 1990; Lim and Kai, 2007; Lasko, 2013; Bozzetti et al., 2015). However, in contrast to the majority of piRNA-related genes (Nishida et al., 2007; Specchia et al., 2008, 2010, 2017; Specchia and Bozzetti, 2009; Bozzetti et al., 2015; Sahin et al., 2016), *belle* seems not to be involved in the *crystal-Stellate* regulatory pathway, since mutant flies did not exhibited Stellate-made crystalline aggregates in the spermatocytes (Supplementary Information and Supplementary Figure S6). This observation well-agrees with the demonstration that *belle ^EY 08943^* mutant does not show an increase of RNA for TEs and repetitive sequences like *Stellate.*

A possible role of BELLE in the piRNA pathway is only starting to be elucidated: our experiments suggest that it might act in maintaining precise levels of TE RNAs, probably regulating the activity of other piRNA components.

The involvement of *belle* in both circadian rhythmicity and piRNA mediated transposon regulation suggests association between these two biological processes. This hypothesis is supported by indirect, though reasonable, observations.

First of all, circadian components have been linked to piRNA factors in the gonads. In fact, the mammalian CLOCK and BMAL1 transcription factors have been associated to the chromatoid body (CB) (Peruquetti et al., 2012), a cytoplasmic electron dense structure in the male germline with similarities to *Drosophila* polar granules and “nuage” of the germ cells (Grivna et al., 2006), where many components of different RNA metabolism pathways, like piRNAs, are located (Tanaka et al., 2000; Kotaja et al., 2006; Nagamori et al., 2011).

Secondly, a piRNA gene plays essential roles in neuronal development and circadian regulation. Our group has recently identified *dFmr1*, a gene with well-characterized functions in development of nervous system (Schenck et al., 2002; Napoli et al., 2008; Aitken and Lorsch, 2012; Doll and Broadie, 2016) and in regulation of circadian rhythmicity (Dockendorff et al., 2002; McBride et al., 2005; Santos et al., 2014), as a piRNA gene playing a crucial role in the gonads, where it ensures fertility and piRNA-regulated genome stability (Bozzetti et al., 2015; Specchia et al., 2017). Nevertheless, a role of this gene in the piRNA-mediated regulation in the nervous system has not been reported yet. *dFmr1* mutants display an altered rhythmicity in both eclosion and locomotor activity, due to an effect on the output factor CREB (cAMP response element binding protein) rather than the molecular oscillator (Dockendorff et al., 2002).

Third, circadian transposon regulation ensures genome integrity during aging. It is well-known that TEs activation induces an age-dependent loss of neuronal functions in *Drosophila* brain (Li et al., 2013). Very recently, a set of putative primary piRNA transcripts fully overlapping transposons in antisense were shown to display a clock-controlled *de novo* oscillation in old flies, raising the hypothesis that circadian piRNA expression could represent a new strategy adopted by the clock to preserve genome integrity during aging (Kuintzle et al., 2017).

A clear involvement of the piRNA pathway in the circadian rhythmicity has not been demonstrated so far. However, we have described an emerging role of *belle* in both circadian rhythmicity and transposon regulation, that leads to the attractive hypothesis that piRNA-mediated regulation could be another level of post-transcriptional control adopted by the clock to ensure the proper rhythmicity.

## Author Contributions

RC, GMM, and MPB conceived and supervised the study. PC, MD, EC, LC, VS, and AP performed experiments and analyzed the data. PC, MD, GMM, RC, VS, and MPB discussed results and wrote the manuscript.

## Conflict of Interest Statement

The authors declare that the research was conducted in the absence of any commercial or financial relationships that could be construed as a potential conflict of interest.
