# Supplementary material for: The RNA Helicase BELLE Is Involved in Circadian Rhythmicity and in Transposons Regulation in Drosophila melanogaster
Source: Front Physiol. 2019 Feb 20;10:133. doi: 10.3389/fphys.2019.00133 (PMC6392097; doi:10.3389/fphys.2019.00133)
Supplement: Supplementary file 1 [file Table_1.DOCX]

TABLE S1: Sequence of primers used for qRT-PCR.

| PRIMER | SEQUENCE |
| --- | --- |
| *bel* upper | 5’ TATGATACGTTTGGGGAACC 3’ |
| *bel* lower | 5’ ACCATCCCAAAGGAGCTATC 3’ |
| *roo* upper | 5’ CGTCTGCAATGTACTGGCTCT 3’ |
| *roo* lower | 5’ CGGCACTCCACTAACTTCTCC 3’ |
| *R1* upper | 5’ TGGCGAAACTTGATGTAGGA 3’ |
| *R1* lower | 5’ GCGGCAAACACTCTCCTTCT 3’ |
| *blood* upper | 5’ CAGCGAATGTCTACCAAGCA 3’ |
| *blood* lower | 5’ TTCTTTTTAGGTGGCTGCTC 3’ |
| *I* upper | 5’ CAATCACAACAACAAAATC 3’ |
| *I* lower | 5’ GGTGTTGGTGTGGTTGGTTG 3’ |
| *ZAM* upper | 5’ TCGTCGCCGCAGGAAACTCTC 3’ |
| *ZAM* lower | 5’ GTGGAGCGACGATTGGAAGAA 3’ |
| *rp49* upper | 5’-ATCGGTTACGGATCGAACAA-3’ |
| *rp49* lower | 5’-GACAATCTCCTTGCGCTTCT-3’ |
