# Supplementary material for: The RNA Helicase BELLE Is Involved in Circadian Rhythmicity and in Transposons Regulation in Drosophila melanogaster
Source: Front Physiol. 2019 Feb 20;10:133. doi: 10.3389/fphys.2019.00133 (PMC6392097; doi:10.3389/fphys.2019.00133)
Supplement: Supplementary file 2 [file Table_2.doc]

Table S2. List of the proteins identified by LC-MS/MS^a^

| Gel  band | Acc. No. | MM (Da) | Protein name | Sequences of the tryptic peptides |
| --- | --- | --- | --- | --- |
| BELLE  (~ 85 KDa)^a^ | Q9VHP0 | 85,371 | ATP-dependent RNA helicase bel | FLVLDEADR  HAIPIIINGR  DSLTLIFVETK  ELATQIFEEAK  SYLLDLLSSIR  QYPLGLVLAPTR  IVEQLNMPPTGQR  SGDCPILVATAVAAR  NICSDLLELLIETK  HVINFDLPSDVEEYVHR  VGSTSENITQTILWVYEPDKR |
| ME31B  (~ 55 KDa) ^a^ | P23128 | 52,539 | Putative ATP-dependent RNA helicase me31b OS=Drosophila melanogaster | LVLDEADK  VQLIIATPGR |
