# Supplementary material for: The RNA Helicase BELLE Is Involved in Circadian Rhythmicity and in Transposons Regulation in Drosophila melanogaster
Source: Front Physiol. 2019 Feb 20;10:133. doi: 10.3389/fphys.2019.00133 (PMC6392097; doi:10.3389/fphys.2019.00133)
Supplement: Supplementary file 3 [file Table_3.DOCX]

**Table S3. Locomotor activity of *belle* Knock-Down flies under LD cycles and DD conditions.**

| **Genotype** | **N**  **Tot** | **N**  **Alive** | **N**  **R** | **%**  **R** | **τ** |  | **SEM** | **MA** | **EA** | **MI** | **±** | **SEM** |
| --- | --- | --- | --- | --- | --- | --- | --- | --- | --- | --- | --- | --- |
| *GMR-Gal4/+;UAS-RNAi-belle* | 64 | 62 | 60 | 96.77 | 23.95 | ± | 0.04 | 96.77 | 100 | 0.205 | **±** | 0.016 |
| *ninaE-Gal4/UAS-RNAi-belle* | 60 | 56 | 53 | 94.64 | 24.02 | ± | 0.05 | 76.79 | 96.43 | 0.125 | **±** | 0.022 |
| *GMR-Gal4/+* | 32 | 32 | 30 | 93.75 | 23.85 | ± | 0.07 | 81.25 | 96.88 | 0.119 | **±** | 0.030 |
| *ninaE-Gal4/+* | 28 | 27 | 24 | 88.89 | 23.97 | ± | 0.05 | 88.89 | 100 | 0.095 | **±** | 0.024 |

R: rhythmic flies. MA: morning anticipation; EA: evening anticipation. MA and EA were detected, fly-by-fly, examining the bout of activity prior to light transitions. MI: Morning Index. The experiments were performed at 23°C.
