# Supplementary material for: The RNA Helicase BELLE Is Involved in Circadian Rhythmicity and in Transposons Regulation in Drosophila melanogaster
Source: Front Physiol. 2019 Feb 20;10:133. doi: 10.3389/fphys.2019.00133 (PMC6392097; doi:10.3389/fphys.2019.00133)
Supplement: Supplementary file 4 [file Data_Sheet_1.PDF]

Supplementary methods

For the analysis of BELLE expression, heads of flies reared in LD cycles for at least 3 days and collected at indicated time points were subjected to protein extraction as described elsewhere (Edery et al., 1994). SDS PAGE was performed in 4-12% Bis-Tris NuPAGE gel (Thermo Fisher Scientifics, Waltham, MA, USA), following manufacturer protocols. Membranes were probed with the anti-BELLE antibody previously described and a mouse anti HSP70 (SIGMA, 1:10000) for loading control. The anti-rabbit and anti-mouse previously described were used as secondary antibodies. Protein levels across time points were compared by densitometry (ImageJ).

Immunofluorescence of Stellate-made crystals

Testes were dissected in Ringer's modified solution (182 mM KCl, 46 mM NaCl, 3 mM CaCl2, 10 mM Tris-HCl pH 7.5). Testes were fixed with methanol, washed with PBST (1Å~ PBS, 1% Triton X-100, 0.5% acetic acid) for 15 min, washed with 1Å~ PBS for 5 min three times and incubated with the polyclonal mouse anti-Ste antibody (1:100) (Bozzetti et al., 1995). Samples were washed with 1X PBS for 5 min three times, incubated 2 h with 1:100 FITC-conjugated anti-mouse-IgG antibody (Jackson) and examined by epifluorescence microscopy (Nikon-Optiphot 2). DAPI was used at 100 ng/ml for the nuclear labelling.

Supplementary results

<u>crystal-Stellate regulation is not altered in belle mutants</u>

The majority of piRNA-related genes exhibit crystalline aggregates in spermatocytes of mutants, as result of the increased expression of the *Stellate* sequences located on the X chromosome, indicating a role for these genes in the *crystal-Stellate* regulation (Nishida et al., 2007; Specchia et al., 2008; Specchia and Bozzetti, 2009; Specchia et al., 2010; Bozzetti et al., 2015; Sahin et al., 2016; Specchia et al., 2017). In order to unravel a possible involvement of *belle* in this regulatory pathway, we searched for the presence of crystals in testes of *belle* mutants: none of them exhibited Stellate-made crystalline aggregates in the spermatocytes (Figure S7) and this observation well agrees with the demonstration that $belle^{EY08943}$ mutant does not show an increase of RNA for TEs and repetitive sequences like *Stellate.* These observations clearly indicate that *belle* "loss of function" mutation does not interfere with the *crystal-Stellate* regulation.

References

Bozzetti, M. P., Massari, S., Finelli, P., Pinna, L. A., Boldyreff, B., Issinger, O.-G., et al. (1995). The Ste locus, a component of the parasitic cry-Ste system of Drosophila melanogaster, encodes a protein that forms crystals in primary spermatocytes and mimics properties of the beta subunit of casein kinase 2. *Proc Natl Acad Sci U S A.* 92, 6067–6071.

Bozzetti, M.P., Specchia, V., Cattenoz, P.B., Laneve, P., Geusa, A., Sahin, H.B., et al. (2015) The Drosophila fragile X mental retardation protein participates in the piRNA pathway. J Cell Sci, 128, 2070-2084. doi: 10.1242/jcs.161810

Edery, I., Zwiebel, L. J., Dembinska, M. E., and Rosbash, M. (1994). Temporal phosphorylation of the Drosophila period protein. *Proc. Natl. Acad. Sci. U. S. A.* 91, 2260–2264. doi:10.1073/pnas.91.6.2260.

Nishida, K.M., Saito, K., Mori, T., Kawamura, Y., Nagami-Okada, T., Inagaki, S., Siomi, H. and Siomi, M.C. (2007) Gene silencing mechanisms mediated by Aubergine piRNA complexes in Drosophila male gonad. *Rna*, 13, 1911-1922. doi: 10.1261/rna.744307

Sahin, H.B., Karatas, O.F., Specchia, V., Tommaso, S.D., Diebold, C., Bozzetti, M.P. and Giangrande, A. (2016) Novel mutants of the aubergine gene. *Fly*, 10, 81-90. doi: 10.1080/19336934.2016.1174355

Specchia, V., Benna, C., Mazzotta, G.M., Piccin, A., Zordan, M.A., Costa, R. et al. (2008) aubergine gene overexpression in somatic tissues of aubergine(sting) mutants interferes with the RNAi pathway of a yellow hairpin dsRNA in Drosophila melanogaster. *Genetics*, 178, 1271-1282. doi: 10.1534/genetics.107.078626

Specchia, V. and Bozzetti, M.P. (2009) Different aubergine alleles confirm the specificity of different RNAi pathways in Drosophila melanogaster. *Fly*, 3, 170-172. PMID: 19242123

Specchia, V., D'Attis, S., Puricella, A. and Bozzetti, M.P. (2017) dFmr1 Plays Roles in Small RNA Pathways of Drosophila melanogaster. *Int J Mol Sci*, 18. doi: 10.3390/ijms18051066

Specchia, V., Piacentini, L., Tritto, P., Fanti, L., D'Alessandro, R., Palumbo, G., et al. (2010) Hsp90 prevents phenotypic variation by suppressing the mutagenic activity of transposons. *Nature*, 463, 662-665. doi: 10.1038/nature08739
