# Supplementary figures and images for: The RNA Helicase BELLE Is Involved in Circadian Rhythmicity and in Transposons Regulation in Drosophila melanogaster
Source: Front Physiol. 2019 Feb 20;10:133. doi: 10.3389/fphys.2019.00133 (PMC6392097; doi:10.3389/fphys.2019.00133)

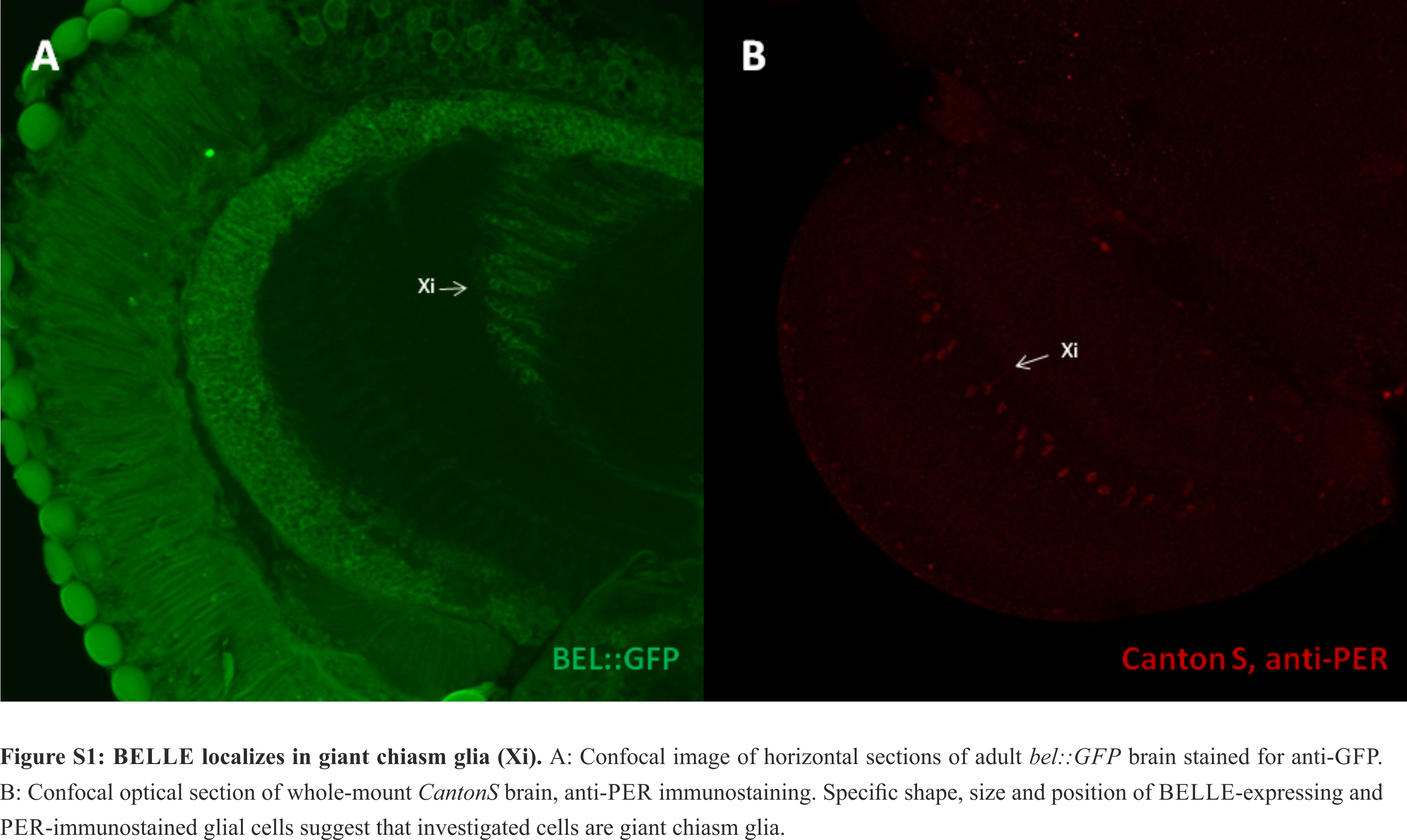

Supplement: Supplementary file 5 [file Image_1.TIF]

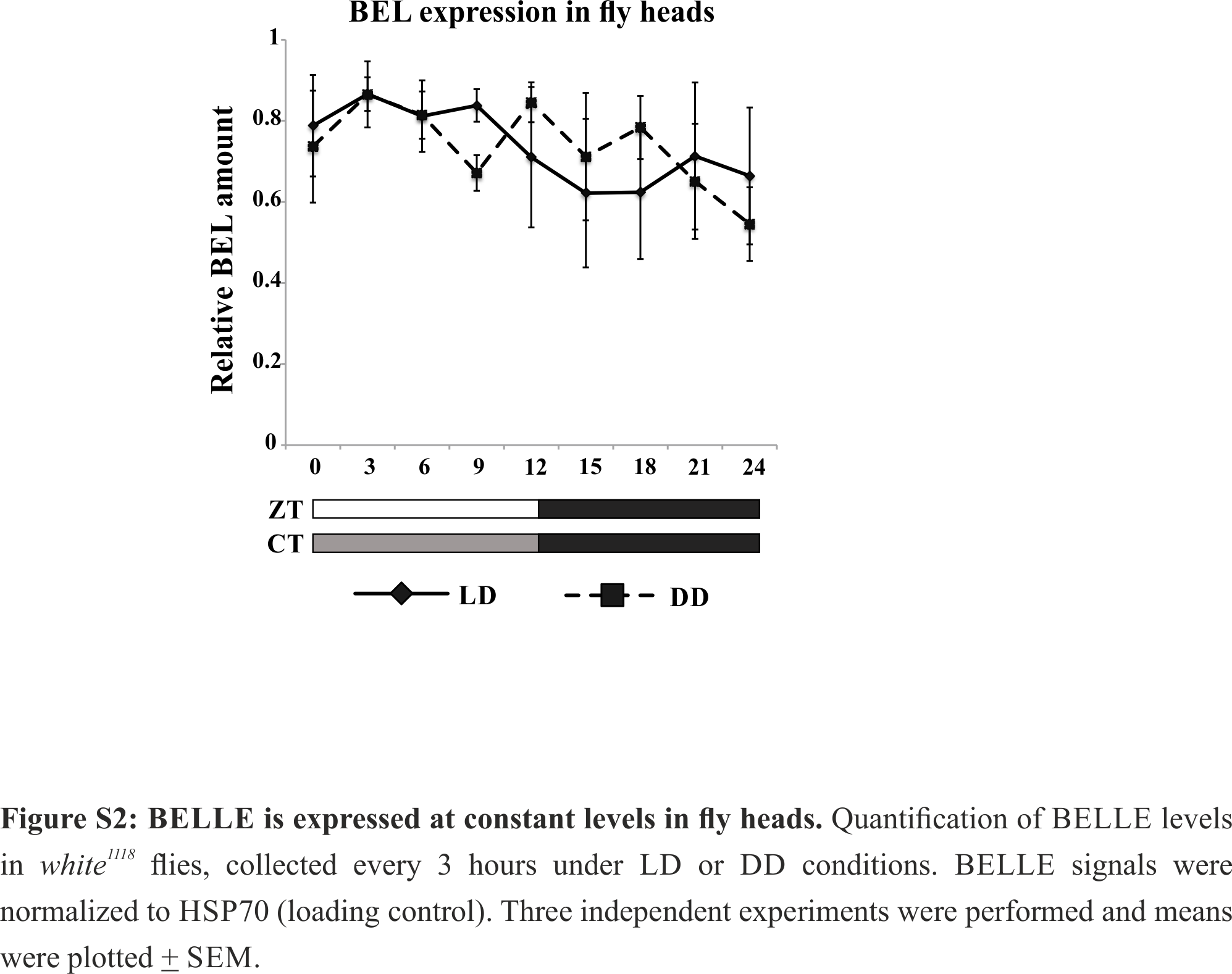

Supplement: Supplementary file 6 [file Image_2.TIF]

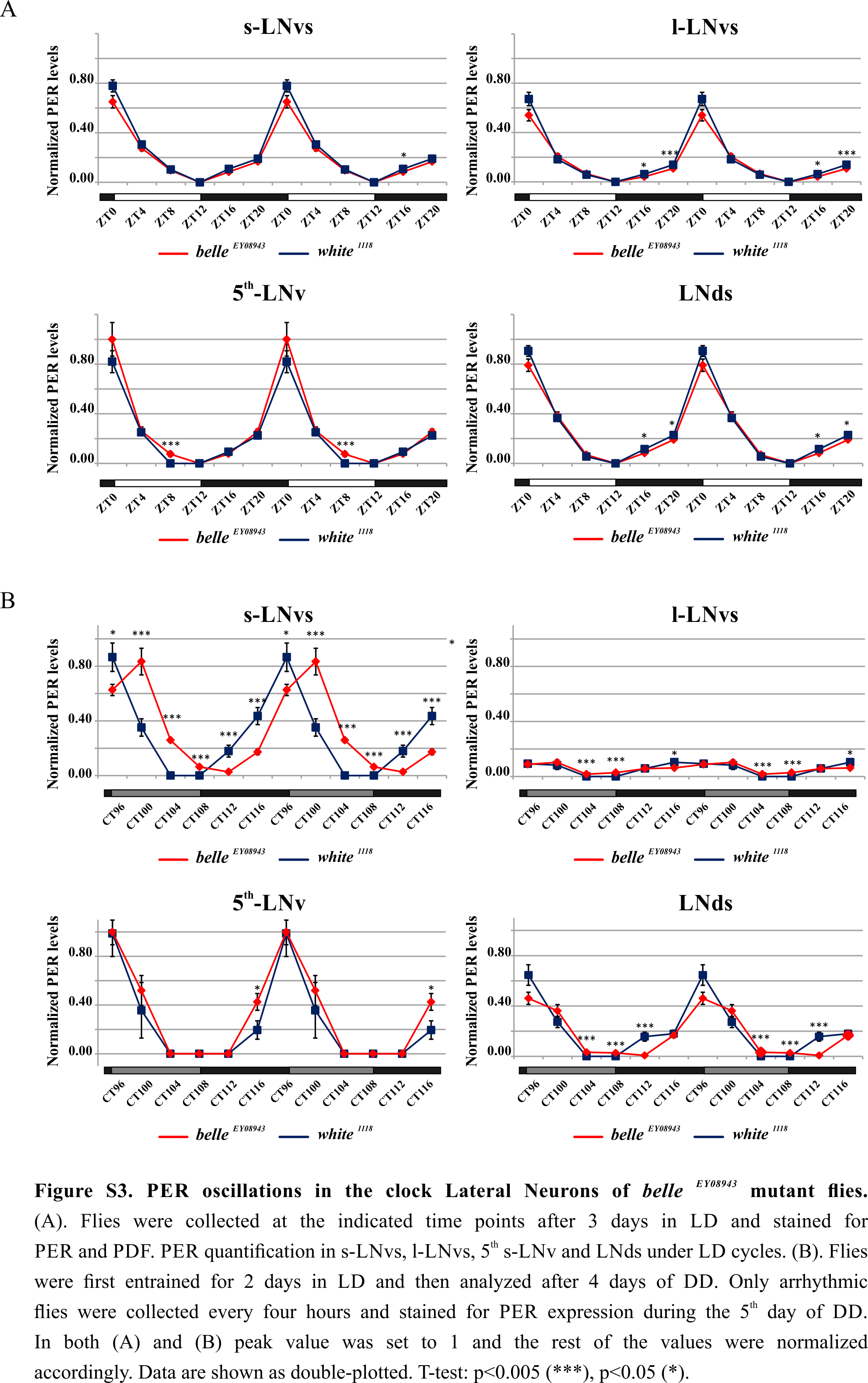

Supplement: Supplementary file 7 [file Image_3.TIF]

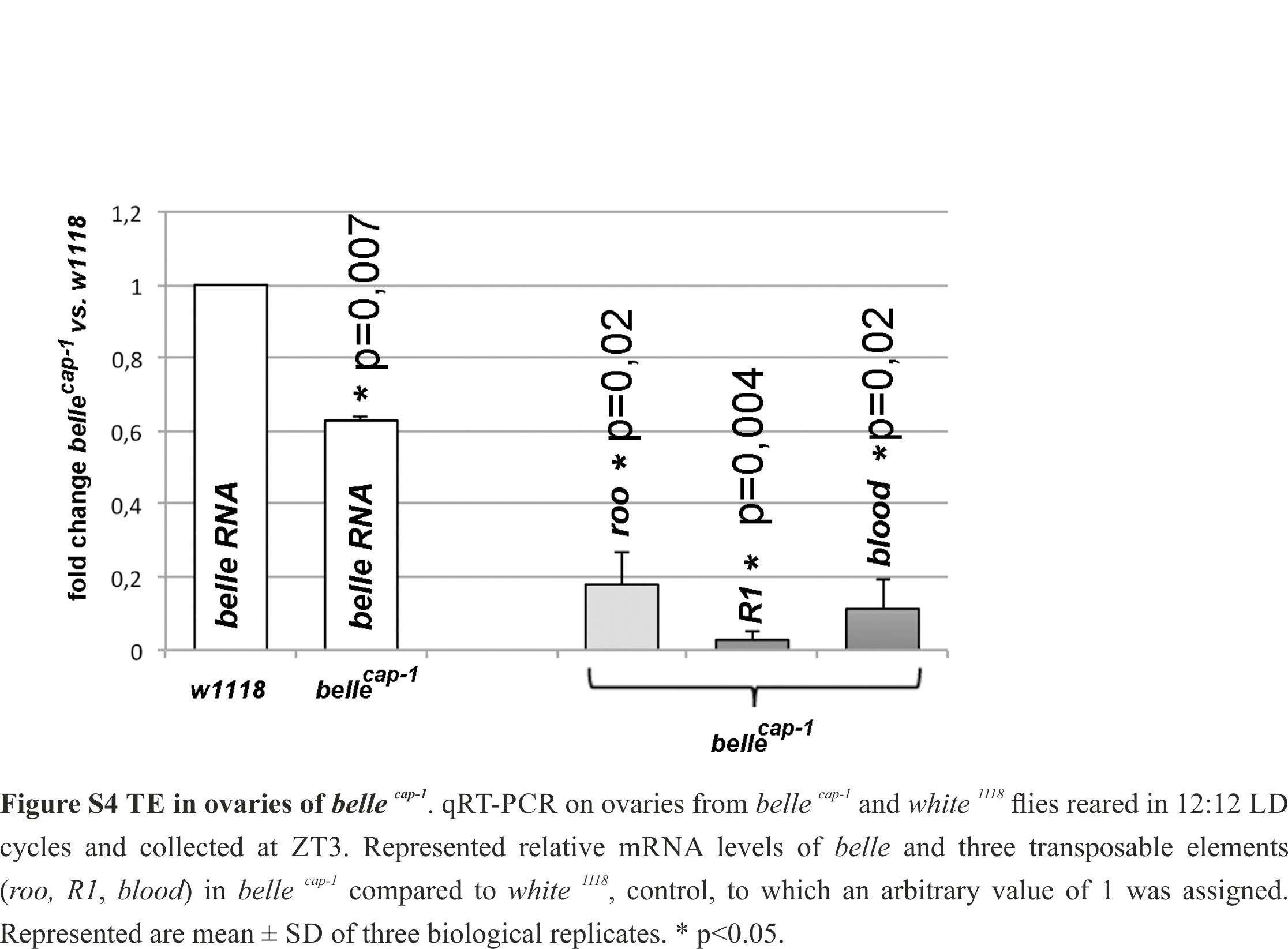

Supplement: Supplementary file 8 [file Image_4.TIF]

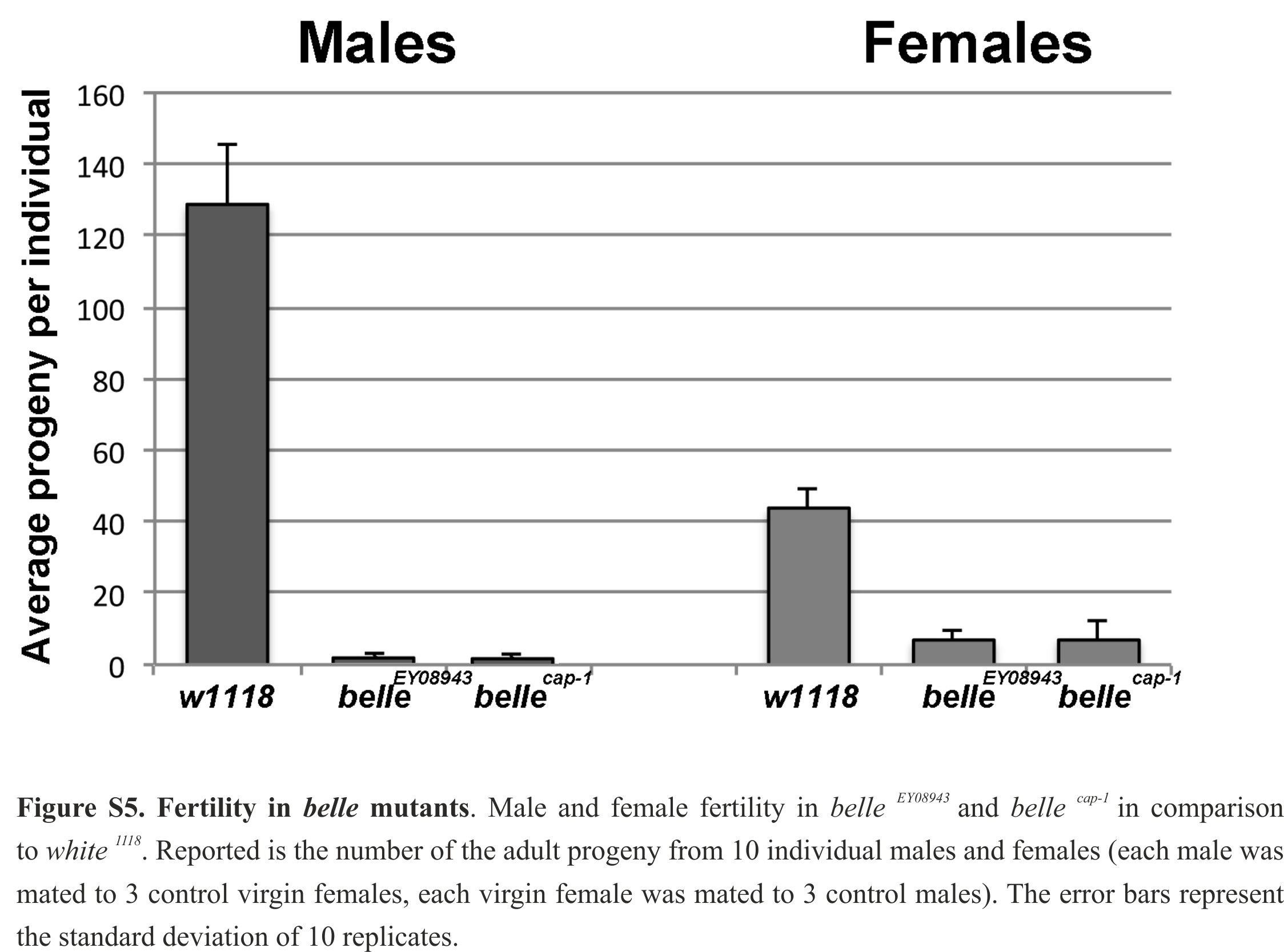

Supplement: Supplementary file 9 [file Image_5.TIF]

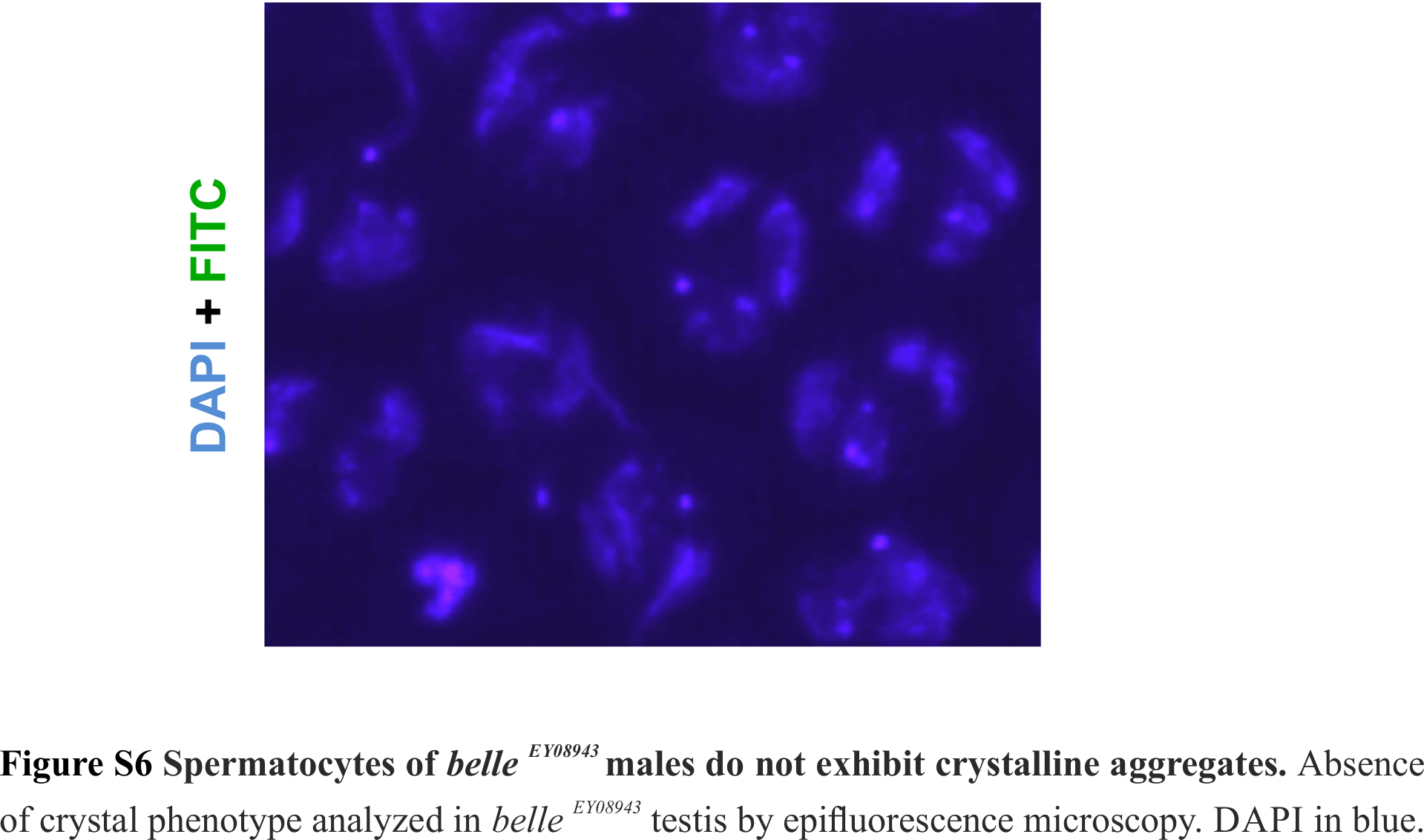

Supplement: Supplementary file 10 [file Image_6.TIF]
